# A hyperspectral deep learning attention model for predicting lettuce chlorophyll content

**DOI:** 10.1186/s13007-024-01148-9

**Published:** 2024-02-03

**Authors:** Ziran Ye, Xiangfeng Tan, Mengdi Dai, Xuting Chen, Yuanxiang Zhong, Yi Zhang, Yunjie Ruan, Dedong Kong

**Affiliations:** 1 Institute of Digital Agriculture, Zhejiang Academy of Agricultural Sciences, Hangzhou, 310,021 Zhejiang China; 2 Hangzhou Institute for Advanced Study, UCAS, Hangzhou, 310,024 China; 3 lnstitute of Agricultural Bio-Environmental Engineering, College of Bio-systems Engineering and Food Science, Zhejiang University, Hangzhou, 310,058 China; 4 Academy of Rural Development, Zhejiang University, Hangzhou, 310,058 China

**Keywords:** Lettuce, Chlorophyll content, Beep learning, Hyperspectral

## Abstract

**Background:**

The phenotypic traits of leaves are the direct reflection of the agronomic traits in the growth process of leafy vegetables, which plays a vital role in the selection of high-quality leafy vegetable varieties. The current image-based phenotypic traits extraction research mainly focuses on the morphological and structural traits of plants or leaves, and there are few studies on the phenotypes of physiological traits of leaves. The current research has developed a deep learning model aimed at predicting the total chlorophyll of greenhouse lettuce directly from the full spectrum of hyperspectral images.

**Results:**

A CNN-based one-dimensional deep learning model with spectral attention module was utilized for the estimate of the total chlorophyll of greenhouse lettuce from the full spectrum of hyperspectral images. Experimental results demonstrate that the deep neural network with spectral attention module outperformed the existing standard approaches, including partial least squares regression (PLSR) and random forest (RF), with an average R^2^ of 0.746 and an average RMSE of 2.018.

**Conclusions:**

This study unveils the capability of leveraging deep attention networks and hyperspectral imaging for estimating lettuce chlorophyll levels. This approach offers a convenient, non-destructive, and effective estimation method for the automatic monitoring and production management of leafy vegetables.

## Background

Lettuce is essential to a healthy human diet. It is a leafy green vegetable that is widely grown and widely consumed, and China holds the title of being the largest lettuce producer in the world [10, 11]. In recent years, there has been a notable rise in the production of lettuce cultivated within controlled environmental systems. In contrast to traditional field cultivation, controlled environment cultivation has emerged as a prevalent method, enabling year-round lettuce and vegetable production irrespective of geographical and climatic limitations [2, 5].

The plant phenotype results from a complex interplay of genetic and environmental factors. It encompasses all the physical, physiological, and biochemical characteristics, including structural traits and composition, as well as the various processes associated with plant growth and fruit development. Green plant leaves engage in the process of photosynthesis, which transforms absorbed solar energy into organic substances [17]. Additionally, they play a vital role in facilitating the exchange of water vapor and carbon dioxide through small openings known as stomata. Furthermore, leaves acquire oxygen through respiration. This entire process collectively generates the essential energy required for plant growth and metabolic activities [15]. The phenotypic traits of leaves serve as indicators of a plant's capacity to respond, adapt, and self-regulate in ever-changing environmental conditions [25]. For leafy vegetable species commonly consumed by humans, such as lettuce, cabbage and various green vegetables, the morphological and physiological traits of leaves can reflect the growth status of leafy vegetables. Chlorophyll is a group of main pigments used for photosynthesis in green plants and is closely related to the physiological state of plants [12, 25]. Monitoring chlorophyll content can reflect the health of plants, and thus the evaluation and prediction of leaf chlorophyll is of great significance for lettuce cultivation.

With the development of computer vision and digital imaging technology, hyperspectral imaging (HSI) has become increasingly feasible. HSI combines machine vision and spectroscopy to furnish concurrent spatial and spectral data pertaining to the observed object [16]. The hypercube data acquired from the HSI sensor comprises complete spectral details for each pixel, in addition to texture or statistical attributes for each wavelength band [13]. HSI has demonstrated significant potential as a non-invasive tool, offering insights into physical morphological, as well as physiological and biochemical properties of plants. Nowadays, this technique has been widely used in plant phenotyping [9]. Guo et al. [7] investigated the quantitative relations between leaf chlorophyll content and three kinds of canopy hyperspectral parameters in tobacco. Zhang et al. [26, 27] employed the partial least squares regression (PLSR) algorithm to establish a prediction model for estimating the relative canopy chlorophyll content of sugar beet using proximal hyperspectral imagery. Rehman et al. [14] proposed a deep learning model utilizing 1-D convolutional neural network (CNN) designed to estimate the relative water content (RWC) of maize plants using mean spectral reflectance. Similarly, Zhou et al. [30] established a sugar content prediction 1-D CNN model for HSI of green plums. In terms of lettuce, Eshkabilov et al. [6] estimated nutrient levels of the lettuce cultivated within a hydroponic tub culture system. using HSI technique along with principal component analysis (PCA) and PLSR method. In a recent investigation by Yu et al. [24], deep learning models were employed to forecast water stress levels in lettuce using hyperspectral data. Several studies have been undertaken to predict parameters such as fresh weight, nitrate, pH value, soluble solid content and pigment content of lettuce using hyperspectral data [6, 18, 28]. Nevertheless, most current proximal hyperspectral-based studies rely on multivariate analysis methods. These methods often entail intricate feature engineering, which require considerable expertise and multiple fine-tunings. Concurrently, deep learning methods like CNNs are capable to automatically extract features from image data, presenting novel opportunities for more streamlined and efficient feature engineering. Therefore, it is necessary to explore and develop methods for estimating chlorophyll content using full hyperspectral spectra with the help of deep learning to mitigate the computational challenges arising from the inherent high dimensionality of hyperspectral data.

The purpose of this study are as follows: (1) To develop a convenient deep learning approach incorporating 1-D CNN, attention modules, and hyperspectral data for estimating the chlorophyll content of lettuce; (2) The effect of the spectral attention module with a gating mechanism for assisting the deep CNN model to learn the importance of the hyperspectral bands was analyzed; (3) Comparing the estimation performance of the developed deep learning model against that of linear partial least squares (PLS) and random forest (RF) regression models, all utilizing the full spectrum.

## Methods

### Plant materials

In the study, 161 seedlings of “1507 lixiang” lettuce variety were planted. Each lettuce seedling grew in a pot containing a substrate mixture of 3:1:1 of peat soil, perlite and vermiculite, which provided a certain amount of base fertilizer. These pots were placed inside an artificial climate chamber (relative humidity 85%) at the Zhejiang Academy of Agricultural Sciences, Hangzhou, China (30°18′N, 120°12′E). During the three single-factor (temperature, light intensity and photoperiod) lettuce growth experiments, imaging was conducted every 7 days after most of the lettuces had grown their fourth leaf, and a total of 478 samples were obtained. The parameter settings and the number of lettuce plants in each treatment group are shown in Table 1. Under professional supervision, lettuce in the climate chamber was watered every 2 or 3 days after planting, and no fertilizer was applied throughout the experiment.

**Table 1:** Plant materials and treatments

Group	Temperature/℃			Light intensity /lux			Photoperiod (light: dark)/hours		
Treatment	22	**25**	28	16,000	20,000	**24,000**	**16: 8**	12: 6	8: 4
Number of plants	18	17	18	18	18	18	18	18	18
Sample size	72	68	72	36	36	36	50	54	54

^*^The bold items indicate the constant values of the other two parameters during the single-parameter experiment

### Determination of chlorophyll content

Measurements were carried out every week after treatment. The biggest fully expanded leaves from each treatment were used for measurements. Leaf total chlorophyll levels were assessed using a chlorophyll meter (SPAD-502; KONICA MINOLTA, Osaka, Japan). Three locations were randomly selected within 50% region from the leaf base to measure the total chlorophyll content of leaves, and the average was calculated.

### Hyperspectral image system

**Fig. 1:**
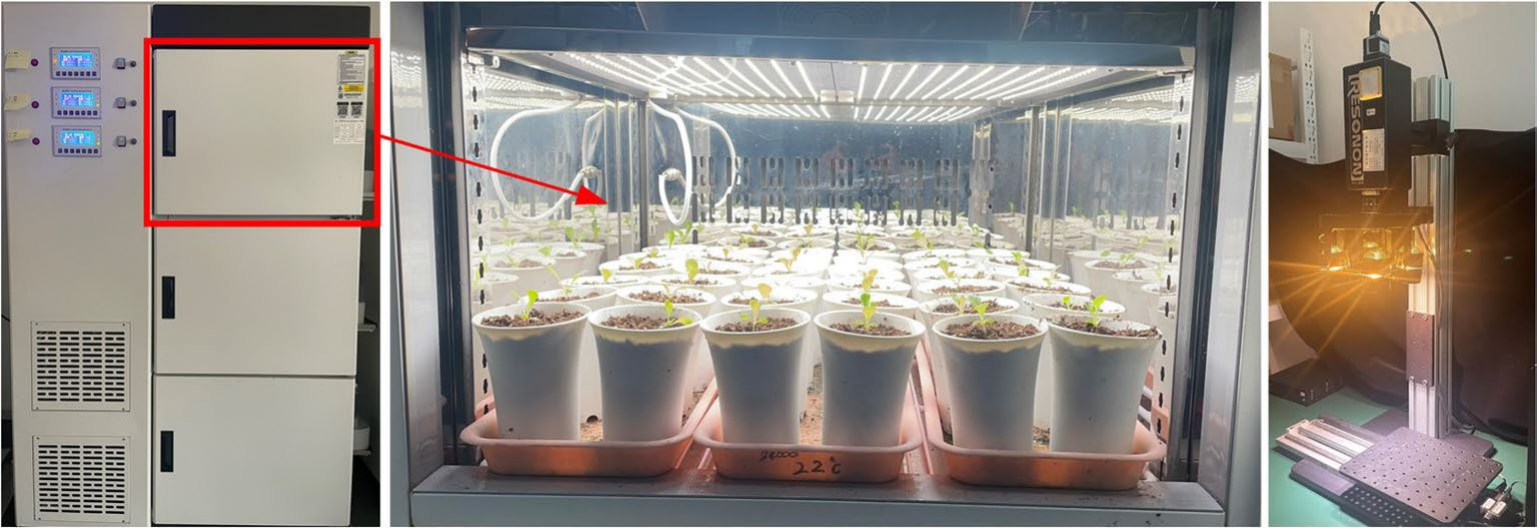
The materials and imaging equipment

A line-scanning hyperspectral imaging system operating in the visible near-infrared (Vis–NIR) range, spanning from 387 to 1003 nm with a bandwidth of 1.3 nm, was utilized for capturing hyperspectral images of lettuce within a controlled, darkroom environment. The Vis–NIR hyperspectral imaging setup comprised a camera (Pika XC2, Bozeman, MT, USA) equipped with a 17 mm focal length lens, four 35-W tungsten halogen lamps as the light source, and a precision displacement platform driven by a stepper motor (Fig. 1). The entire system was under the command of a computer fitted with compatible data acquisition software. The camera was positioned at 0.65 m from the platform. Before image collection, the system was preheated for 30 min in advance to stabilize the light source.

### Hyperspectral image acquisition and calibration, processing

Each individual lettuce was positioned on a displacement platform and transported to the hyperspectral camera’s field of view using a slider. Before capturing hyperspectral images, the light source of the hyperspectral camera was preheated. Subsequently, the parameters of the hyperspectral camera, such as focal length, exposure time, and gain and scanning speed were adjusted using data acquisition software. Hyperspectral images of lettuce from a top-down perspective were captured within the wavelength range of 387–1003 nm. Due to variations in lettuce size at different stages, the size of the hyperspectral images of lettuce ranged from [1600 × 1000] to [1600 × 1400] pixels across 231 bands.

The raw hyperspectral images were preprocessed to mitigate the impact of sensor noise, including dark current, and to correct for non-uniform spectral responses due to varying illumination across different spectral bands. A white plastic panel was utilized as a reference to calibrate the illumination across the images. The dark references were collected to remove the dark noise originating from the hyperspectral sensor. Specifically, this was done by turning off all light sources in the dark room and covering the lens with its opaque black lens cap. The reflectance of HSI was then calibrated using the following equation:

1 $${\text{Reflectance}}=\frac{{I}_{raw}-{I}_{d}}{{I}_{w}-{I}_{d}}$$

where ${I}_{raw}$ is the raw hyperspectral image, ${I}_{d}$ is the dark reference image obtained by covering the camera lens, ${I}_{w}$ is the white reference image. The white reference image and dark reference image were recorded, respectively, under the same experimental conditions as the lettuce image acquisition. The calibration of raw hyperspectral images was performed using the data acquisition software. To mitigate the influence of high noise levels observed at the initial and concluding segments of the spectrum, the spectral range considered for subsequent analysis was confined to 437 − 919 nm for hyperspectral data, encompassing 180 bands.

Image processing involves eliminating the background information from the hyperspectral image while retaining the information related to the object. An 8-bit color image was synthesized using a combination of R, G, and B bands in 16-bit hyperspectral images, and the Otsu threshold segmentation method and morphological processing were used to create a mask for green color and remove the background. The foreground pixels belonging to lettuce were extracted by pixel-by-pixel multiplication of the binary mask with the hyperspectral image. The segmented lettuce region was defined as the region of Interest (ROI). The mean spectral reflectance for further analysis was calculated by averaging the reflectance values of all pixels in each band within the ROI and normalizing the average spectrum to the 0–1 range with Min–Max Scaling.

### Models for predicting chlorophyll content of lettuce

The spectral reflectance of plants carries abundant information about their growth and health, encompassing various physiological and biochemical characteristics. Building upon prior research [14, 24], this study employs the Inception module in deep learning. This model takes the mean spectral reflectance as its input and generates estimations of total chlorophyll levels in lettuce. While deep networks based on the Inception module have previously been utilized to predict water stress in maize leaves and lettuce, there is limited existing research on their application for predicting total chlorophyll content in lettuce.

**Fig. 2:**
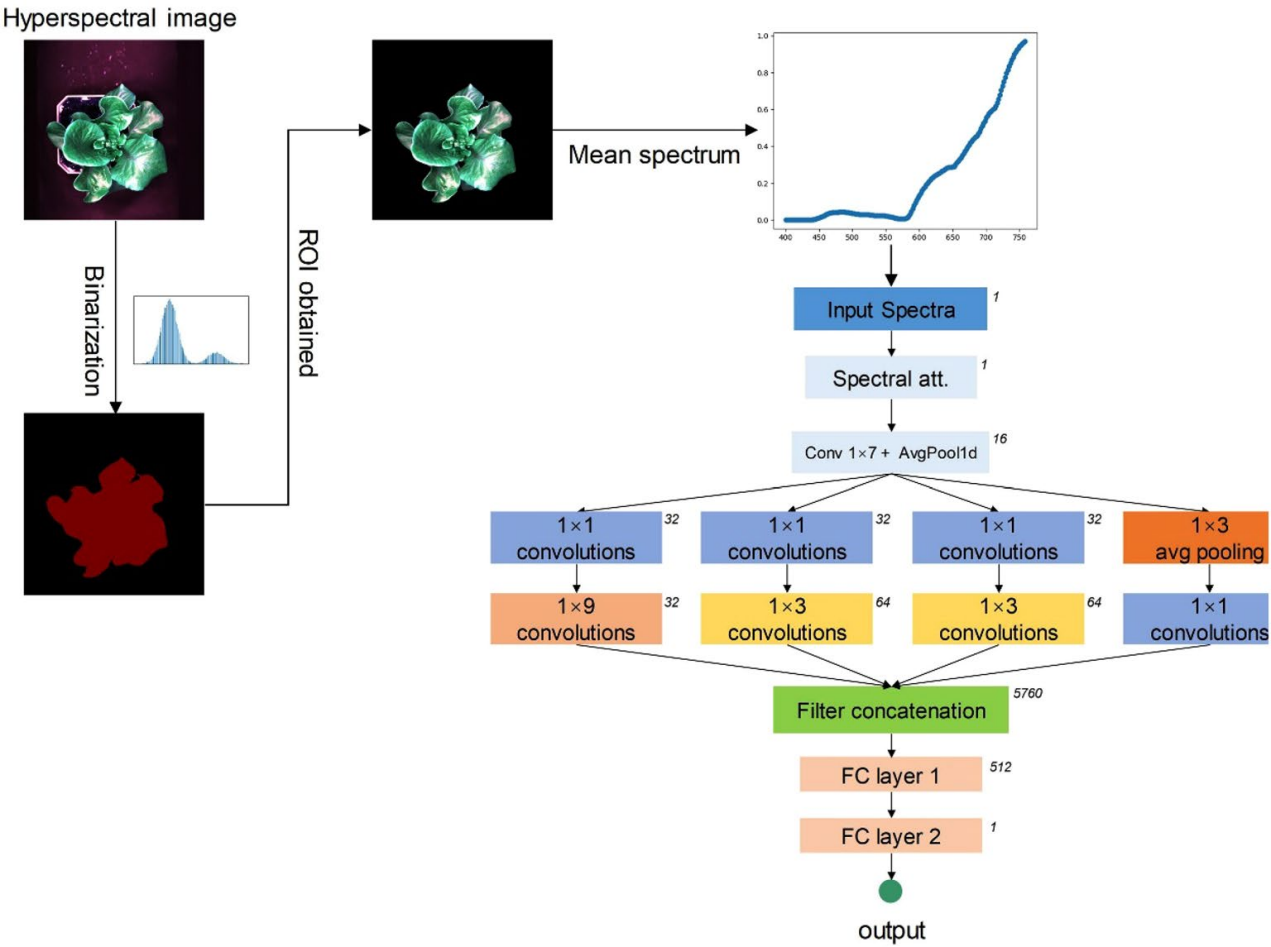
Flowchart for predicting chlorophyll in lettuce using the hyperspectral image and the developed 1-D CNN model

As shown in Fig. 2, the inception-like model used in the study included the input layer, spectral attention module, a convolutional layer and pooling layer, and an inception module followed by two fully connected layers. The convolution layer with stride 2 and the pooling layer with stride 3 performed downsampling after convolving the mean spectral reflectance, resulting in an output feature map of 1/6 size compared to the input. The Inception module was modified to accept 1-D feature maps. The Inception module consists of three parallel convolutional submodules with varying kernel sizes (1 × n, n = 1, 3, 5), along with an average pooling layer. After concatenating the four-branch feature maps generated by the inception module, the estimated value of the total chlorophyll was output through two fully connected layers, where the activation function ReLU was applied between the two fully connected layers.

**Fig. 3:**
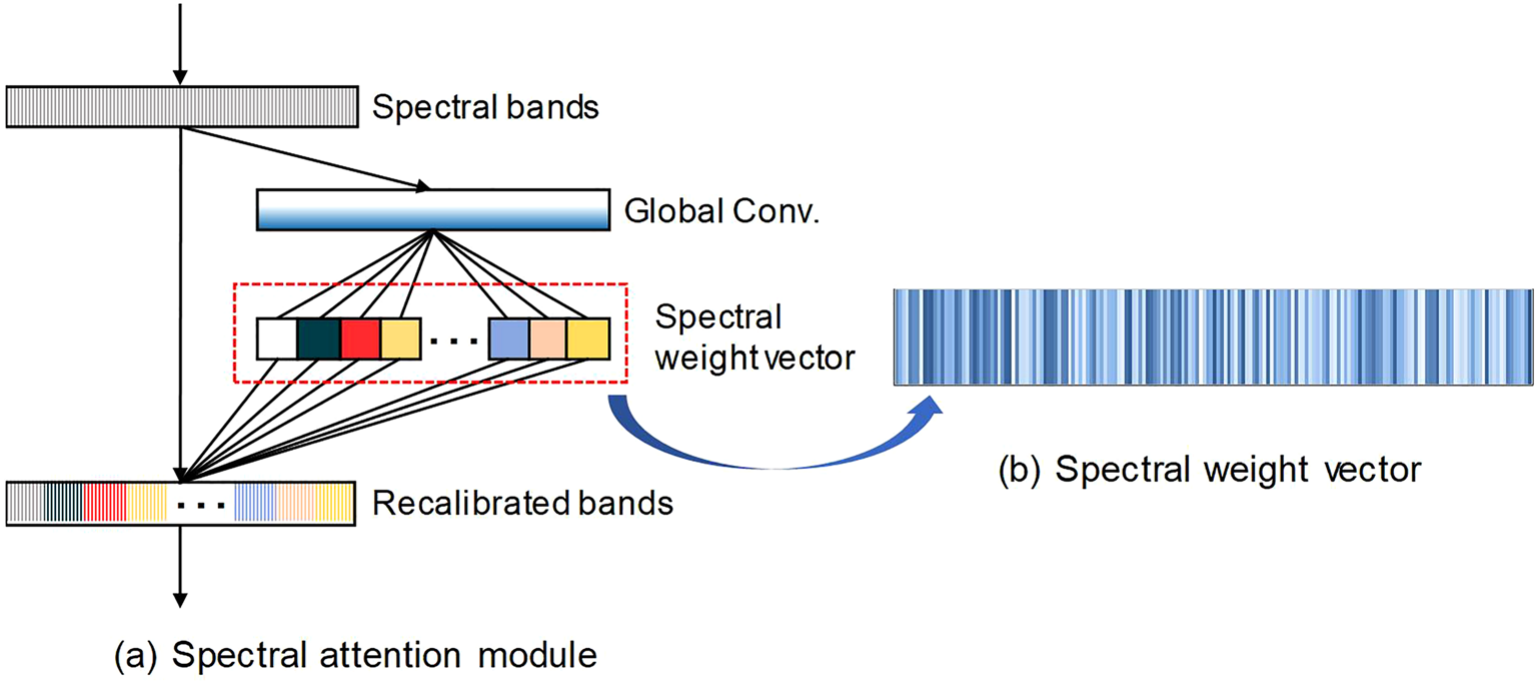
Schematic of the spectral attention module. **a** the spectral weight vector is used to reweight the spectral bands. **b** the real attention value vector from the spectral attention block. The deeper blue indicates the higher weight value

The complexity of high-dimensional spectra in HSI poses a significant hurdle in both data interpretation and practical utilization. However, the process of wavelength selection offers an effective means of mitigating this challenge by reducing the dimensionality of spectral data while preserving critical information [29]. Hence, there is a need in the spectral regression network to consolidate the spectral information from various bands while distinguishing them. In addition, we hope that this spectra module to be task-driven and able to learn adaptively in an end-to-end spectral regression network. For this purpose, a spectral attention module with a gating mechanism was employed to assess the importance of various spectral bands and recalibrate them (Fig. 3). Inspired by the “squeeze and excitation” networks [8], the “squeeze and excitation” block (SE block) was employed to learn the spectra information and recalibrate channel-wise responses adaptively. This spectral attention module was placed after the input and before the first convolutional layer. After inputting the mean spectrum into the module, a vector was generated using global average pooling to serve as channel-wise descriptors. Following this, two fully connected layers were applied to this vector to capture nonlinear interactions among the channels. Subsequently, the processed vector was activated by the sigmoid function to generate a weight vector, which acted as a scaling factor for the various spectral bands. Therefore, the model used in this study was built by the full spectrum.

### Data augmentation and training strategy

In order to augment the spectral data, the offset and slope of the spectral data were changed randomly in a small range. The offset was varied within a range of ± 0.10 times the standard deviation of the spectral curve, and the slope was randomly adjusted to values uniformly distributed between 0.95 to 1.05.

In a fivefold cross-validation, the dataset of 478 images was divided into 5 equal sections. Each section was used as the validation set once, with the remaining parts used for training. This process was repeated for 5 times. This folding technique maximizes the use of limited data, ensuring each training process utilizes a distinct set, preventing bias, and mitigating the risk of overfitting.

During the training phase, the Adam optimizer was employed to update the parameters of the deep CNN. Initially, the learning rate was configured at 0.0001, with a batch size of 6. To assess the model's performance, the mean squared error (MSE) with the L2 norm regularization was utilized as the regression loss function during training. To manage the training process effectively, we implemented a cosine annealing strategy for learning rate adjustments. Specially, the total number of training epoch was set to 500 and the learning rate was reset every 200 epochs.

### Comparison with other standard approaches

PLSR is a widely used method for multivariate data analysis. PLSR simplifies the association between multiple variables by mapping them onto a set of orthogonal feature vectors [19]. This approach is often applied to the detection of plant physiological phenotypes, mainly when using multispectral or hyperspectral images [1].

Random forests (RF) is an ensemble learning method that integrates multiple decision trees by means of bagging [3]. This is also one of the common techniques used for plant physiological component detection. Previous studies have demonstrated that it shows good performance in plant growth traits estimation, which can achieve an accuracy comparable to that obtained using SVM [23, 28].

Grid search was used to optimize the parameters of the above model, which included the key parameter ‘number of components’ of PLSR and the key parameter ‘number of trees’ of RF. Regarding the features selected in PLSR and RF models, two feature selection scenarios were considered: (1) full hyperspectral spectra; (2) sensitive spectral wavelength.

### Metrics for model evaluation

The model’s performance was assessed through the computation of the following metrics: root mean square errors (RMSE), normalized root mean square error (NRMSE) and the correlation coefficient of determination (R^2^). Metrics are defined as follows:

2 $${\text{RMSE}}=\sqrt{\frac{1}{n}\sum_{i=1}^{n}{({y}_{i}-{k}_{i})}^{2}}$$

3 $${\text{NRMSE}}=\frac{\sqrt{\frac{1}{n}\sum_{i=1}^{n}{({y}_{i}-{k}_{i})}^{2}}}{\overline{y} }$$

4 $${R}^{2}=1-\frac{\sum_{i=1}^{n}{({y}_{i}-{k}_{i})}^{2}}{\sum_{i=1}^{n}{({y}_{i}-\overline{y })}^{2}}$$

where n is the number of samples, ${k}_{i}$ is the *i-th* predicted trait, ${y}_{i}$ is the *i-th* ground truth trait, $\overline{y }$ is the average of ground truth.

## Results and analysis

### Estimation results of the developed model

**Fig. 4:**
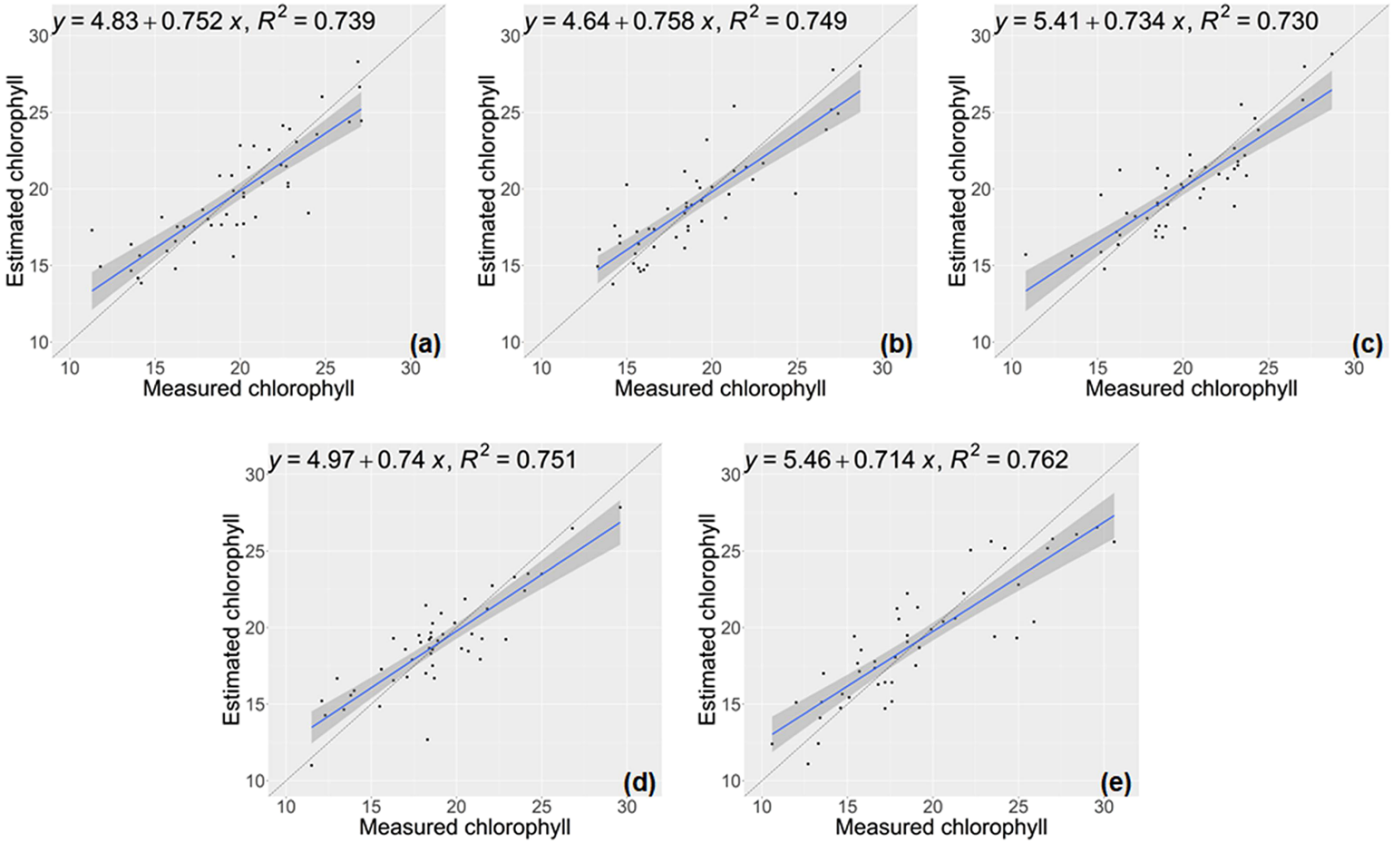
Chlorophyll estimation results using the proposed model. **a**–**e** displays the results from cross-validation

The performance of the model developed for estimating the chlorophyll trait of lettuce on the test sets, as evaluated in a five-fold cross-validation, is presented in Table 2 and illustrated in Fig. 4. The results demonstrated that there was a robust correlation between the chlorophyll values obtained through direct measurements and the predictions generated by our CNN-based model. For chlorophyll trait, the regression model exhibited good estimation capabilities, achieving an average R^2^ of 0.746 and an average RMSE of 2.018.

**Table 2:** Regression error statistics for chlorophyll traits using the proposed method

Number of test sets	RMSE ↓	NRMSE ↓	R^2^ ↑
1	2.035	0.1037	0.739
2	1.960	0.1036	0.749
3	1.870	0.0935	0.730
4	1.868	0.0993	0.751
5	2.358	0.1236	0.762
Average	2.018	0.1047	0.746

### Performance Comparison with standard estimation methods

**Fig. 5:**
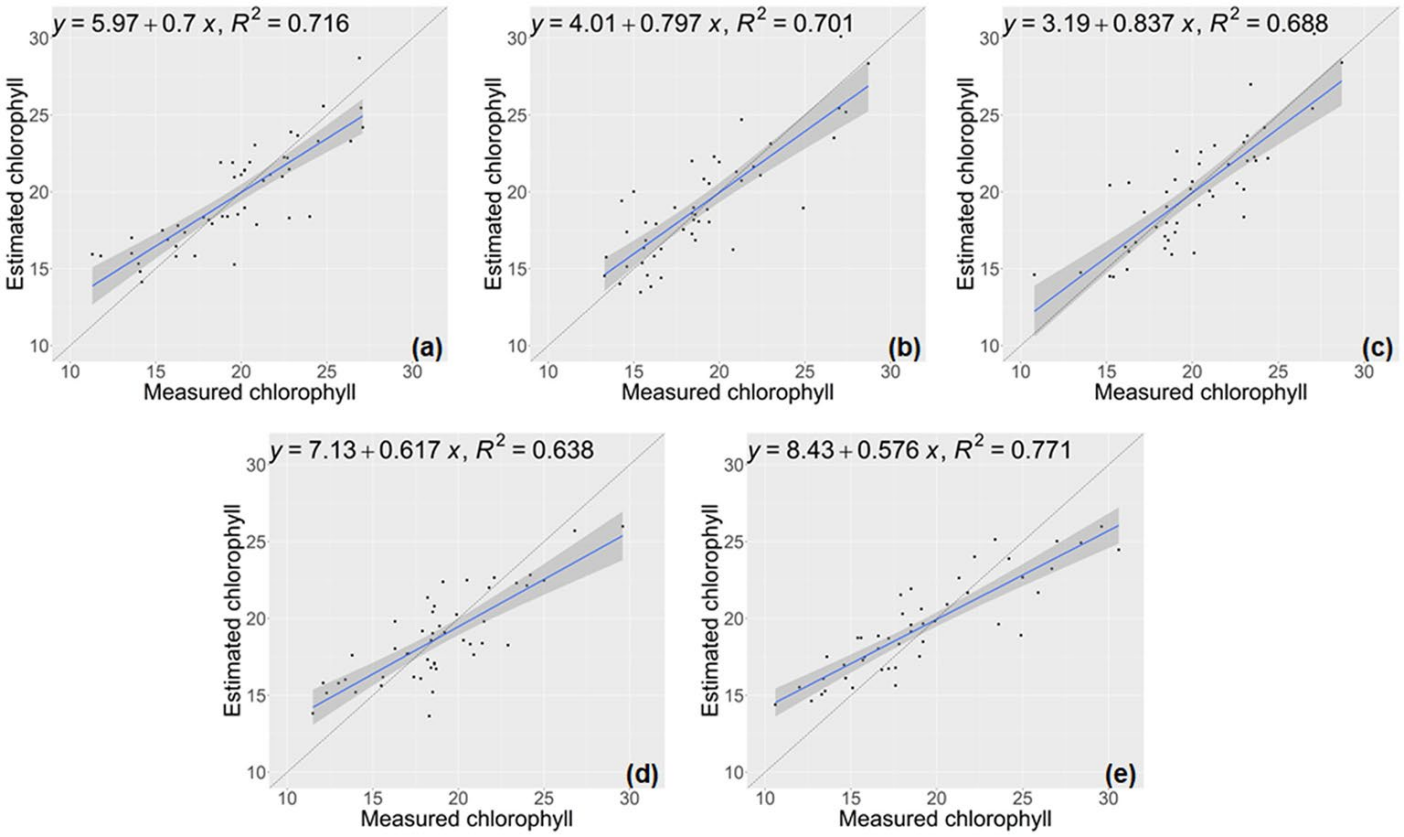
Chlorophyll estimation results using PLSR model. **a**–**e** displays the results from cross-validation

The PLSR model used for comparison was constructed using the entire spectrum to predict chlorophyll content of lettuce. The performance metrics of the PLSR model are detailed in Table 3 and illustrated in Fig. 5, demonstrating an average R^2^ of 0.703 and an average RMSE of 2.247. Overall, the PLSR model utilizing the full spectrum exhibited comparatively lower predictive capacity when contrasted with the deep CNN model.

**Table 3:** Regression error statistics for chlorophyll traits using PLSR

Number of test sets	RMSE ↓	NRMSE ↓	R^2^ ↑
1	2.126	0.1084	0.716
2	2.191	0.1159	0.701
3	2.107	0.1054	0.688
4	2.255	0.1198	0.638
5	2.555	0.1340	0.771
Average	2.247	0.1167	0.703

**Fig. 6:**
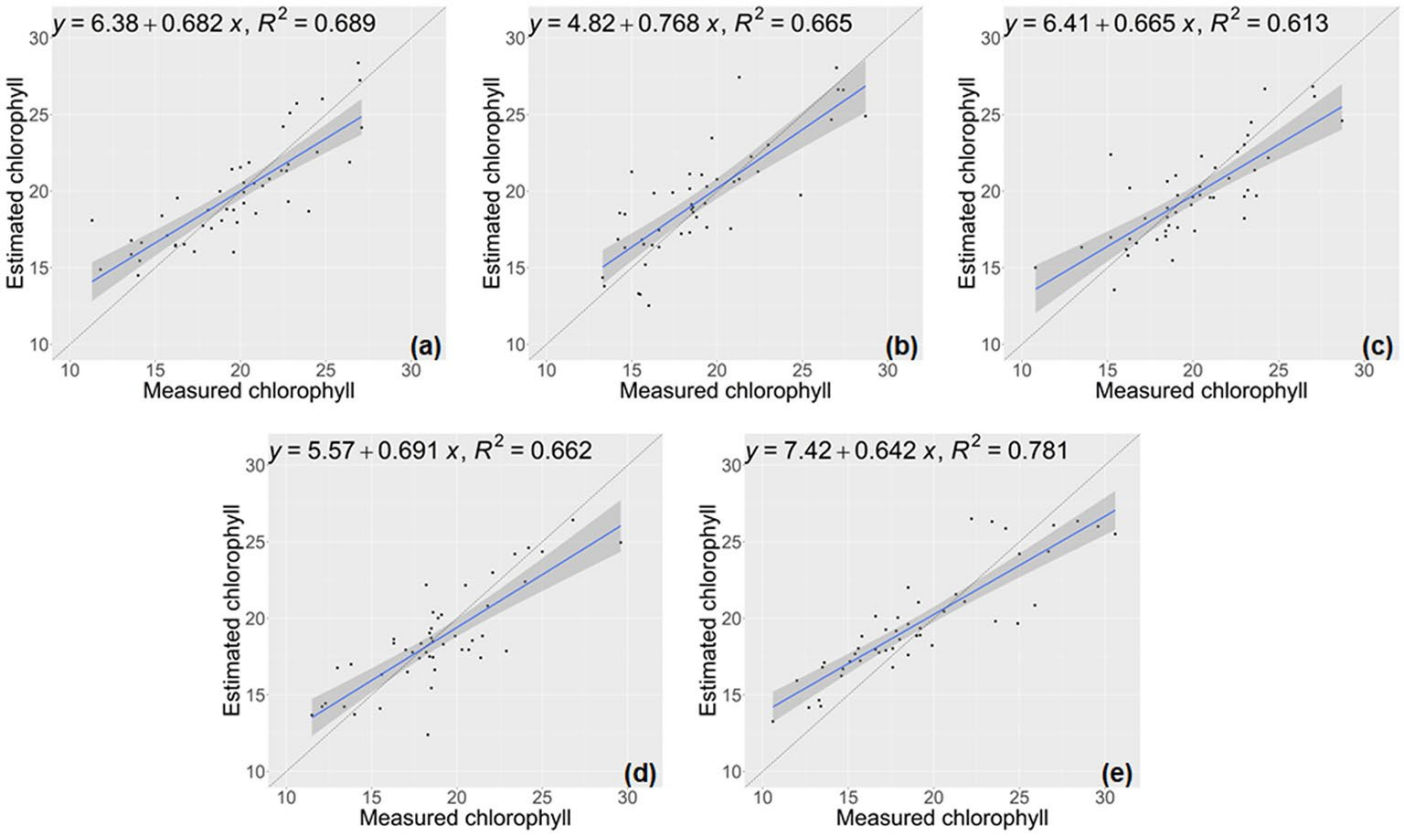
Chlorophyll estimation results using RF model. **a**–**e** displays the results from cross-validation

Similar to the PLSR method, the RF model using the complete lettuce spectrum (Table 4). Through a grid search, the number of trees was determined to be set at 1500. While the RF model achieved an impressive R^2^ score of 0.781 on the fifth test set (Fig. 6.e), the overall performance of RF for chlorophyll estimation yielded an average R^2^ of 0.682, accompanied by an average RMSE of 2.297. This suggests that the RF regression model based on the full spectrum is less robust than the deep model developed in this work.

**Table 4:** Regression error statistics for chlorophyll traits using RF

Number of test sets	RMSE ↓	NRMSE ↓	R^2^ ↑
1	2.228	0.1136	0.689
2	2.356	0.1246	0.665
3	2.266	0.1133	0.613
4	2.191	0.1164	0.662
5	2.443	0.1281	0.781
Average	2.297	0.1192	0.682

### Improvement of attention module on the deep learning model

The spectral attention module based on SE block was incorporated to boost the performance of the 1-D CNN network. Experiments were conducted to investigate the impact of the spectral attention module. As depicted in Table 5, when applying the 1-D inception network without the attention module for chlorophyll estimation, the average R^2^ was inferior to that achieved by the developed model (as shown in Table 2). These results imply that the recalibrated spectra offer improved discrimination. In essence, the spectral attention module based on SE block aids the base network in discerning the most critical spectral bands.

**Table 5:** Regression error statistics of the 1-D inception network without attention module for chlorophyll traits

Number of test sets	RMSE ↓	NRMSE ↓	R^2^ ↑
1	2.081	0.1061	0.727
2	1.994	0.1055	0.740
3	1.988	0.0994	0.693
4	1.940	0.1031	0.731
5	2.391	0.1254	0.752
Average	2.079	0.1079	0.729

### Comparison with regression models with wavelength selection

Spectral regression models with wavelength selection were also involved in the comparison. In terms of wavelength selection algorithms, successive projection algorithm (SPA) was employed for multivariate calibration. This method generated a series of wavelength subsets by iteratively adding one wavelength at a time based on its contribution to the calibration model. SPA has been demonstrated to be an effective strategy for simplifying models (Juanjuan [4, 26, 27]. The subset with the least redundancy and the best performance is selected in the process [20]. For hyperspectral data in this study, the number of variables selected by SPA ranges from 3 to 15 wavelengths because of the differences in the partitioning of the data set in cross-validation. It is worth noting that in each cross-validation, the wavelengths used by PLSR and RF are the same.

Table 6 and Table 7 showed that regression models, constructed based on the optimal bands which selected by SPA, have lower performance than the corresponding regression models constructed directly using the full spectral samples. Instead of using the average spectral reflectance, the first derivative of the reflectance (FDR) data was employed as the input for the machine learning regression model (Table 8 and Table 9). The combination of wavelength selection algorithms with appropriate data preprocessing improves the performance of machine learning models to some extent. However, it still lags the 1-D CNN based deep regression network developed in this work (Table 2).

**Table 6:** Regression error statistics for chlorophyll traits using PLSR (with SPA)

Number of test sets	RMSE ↓	NRMSE ↓	R^2^ ↑
1	2.280	0.1162	0.673
2	2.338	0.1236	0.642
3	2.181	0.1091	0.632
4	2.422	0.1287	0.581
5	2.788	0.1462	0.663
Average	2.402	0.1248	0.638

**Table 7:** Regression error statistics for chlorophyll traits using RF (with SPA)

Number of test sets	RMSE ↓	NRMSE ↓	R^2^ ↑
1	2.194	0.1119	0.697
2	2.463	0.1303	0.603
3	2.310	0.1155	0.587
4	2.418	0.1284	0.583
5	2.837	0.1487	0.651
Average	2.444	0.1270	0.624

**Table 8:** Regression error statistics for chlorophyll traits using PLSR (with SPA + FDR)

Number of test sets	RMSE ↓	NRMSE ↓	R^2^ ↑
1	2.422	0.1235	0.630
2	2.184	0.1155	0.688
3	2.044	0.1022	0.676
4	2.267	0.1204	0.633
5	2.440	0.1279	0.742
Average	2.271	0.1179	0.674

**Table 9:** Regression error statistics for chlorophyll traits using RF (with SPA + FDR)

Number of test sets	RMSE ↓	NRMSE ↓	R^2^ ↑
1	2.249	0.1146	0.681
2	2.133	0.1128	0.702
3	2.098	0.1050	0.659
4	2.661	0.1414	0.494
5	2.567	0.1346	0.714
Average	2.342	0.1217	0.650

## Discussion

To a considerable extent, the core of crop sciences lies in the understanding of how agronomic traits are either selected by breeders or regulated by agronomists [22]. The advancement of plant phenotyping techniques has enabled the successful utilization of image-based methods for acquiring phenotypic data pertaining to both morphology and physiology. In contrast to the considerable focus on morphological traits like leaf senescence, plant height, and growth period, there is a noticeable gap in the capacity for precisely phenotyping physiological traits, which falls short of current demands. As mentioned above, existing studies using hyperspectral imaging to estimate chlorophyll traits in lettuce leaves are dominated by machine learning, and the performance of deep learning techniques to estimate chlorophyll traits of lettuce leaves still needs to be investigated. In this study, we developed a method to nondestructively estimate the chlorophyll of lettuce leaves using hyperspectral techniques and deep learning. In general, when dealing with a large amount of hyperspectral image spectral data, the selection of feature wavelengths is an essential step, through which the redundancy of data can be reduced [21]. Instead of using the traditional wavelength selection algorithm, this study uses a spectral attention module based on a gated mechanism to analyze and dynamically weight the importance of the full spectral bands. The benefit of this is that the spectral attention module can be more easily embedded into existing deep networks and learn the weights of spectral bands based on samples during model building, which implicitly completes the process of wavelength selection in a data-driven manner. When compared to PLSR and RF estimators, the CNN model developed for chlorophyll traits exhibited superior estimation accuracy in this study. These results underscore the strengths of the CNN model in autonomously learning task-oriented features from hyperspectral image data. In particular, the spectral attention module can help CNN models cope with the challenges of the high dimensionality of full hyperspectral data.

Due to the high cost and complex image processing process of hyperspectral imaging systems, the application of hyperspectral imaging systems in controlled environments is mostly in scientific research or project demonstrations rather than practical production in China. Therefore, we plan to design a leafy vegetable transmission device and an automatic acquisition, calibration and processing flow of hyperspectral data for leafy vegetable production scenarios in the controlled environment, pending further research.

Despite the limited scope of utilizing data exclusively from a single lettuce variety under single-factor experimental conditions without additional fertilizer, the results validate the robustness of the deep learning approach developed in this paper for the non-destructive estimation of chlorophyll content in lettuce plants of the specific variety under various treatment conditions (temperature, light intensity, photoperiod). This finding opens the possibility of further exploration of the developed spectral attention CNN framework for applications such as estimating chlorophyll using hyperspectral images of different varieties of lettuce or other leafy crops. Consequently, forthcoming research endeavors can expand the dataset by gathering hyperspectral samples from a wider variety of leafy vegetable species, diverse treatment conditions and management practices. This will not only bolster the model's ability to generalize but also allow for a more comprehensive exploration of how different leafy vegetable species, environmental treatments and management practices influence the performance of the model.

## Conclusions

In this study, we have engineered a one-dimensional deep learning model based on CNNs specifically designed for predicting the total chlorophyll of a single variety of greenhouse lettuce from the mean spectral reflectance. Our model incorporates a customized 1-D Inception module as its core architecture, enabling the effective extraction of multi-scale spectral features. Furthermore, a spectral attention module has been introduced to assess the significance of different hyperspectral bands. This module performs dynamic band-wise recalibration tailored to specific tasks, substantially enhancing the network's representational capacity. What sets the developed approach apart is its data-driven spectral attention module, which allows our 1-D CNN model to harness the complete spectral information without necessitating additional wavelength selection or dimensionality reduction, allowing end-to-end predictions. This not only simplifies the methodology but also makes it more practical for real-world applications. To validate our model's performance, comprehensive comparisons with two standard methods (PLSR and RF) were conducted on a dataset consisting of hyperspectral imagery of lettuce cultivated under various treatments. The quantitative analysis demonstrates that the inception model employed in this study achieved the lowest RMSE and superior R^2^ values on fivefold cross-validation, outperforming existing standard methods.

Based on the comparative evaluation performed in this research, it can be inferred that the deep model utilized in this work shows significant potential in enhancing total chlorophyll content estimation using hyperspectral images of a single lettuce variety grown in a controlled environment. This advancement holds particular promise for high-throughput plant phenotyping applications, aiding in the automation of leafy vegetables monitoring and production management.

## Data Availability

The datasets used and/or analysed during the current study are available from the corresponding author on reasonable request.
